# Enhanced photo-fermentative H_2_ production using *Rhodobacter sphaeroides* by ethanol addition and analysis of soluble microbial products

**DOI:** 10.1186/1754-6834-7-79

**Published:** 2014-05-27

**Authors:** Dong-Hoon Kim, Ji-Hye Lee, Seoktae Kang, Patrick C Hallenbeck, Eui-Jin Kim, Jeong K Lee, Mi-Sun Kim

**Affiliations:** 1 Biomass and Waste Energy Laboratory, Korea Institute of Energy Research, 152 Gajeong-ro, Daejeon, Yuseong-gu 305-343, Republic of Korea; 2 Department of Civil Engineering, Kyung Hee University, 1732 Deokyoungdaero, Yongin, Giheung, Gyeonggi-do 446-701, Republic of Korea; 3 Departement of Microbiology and Immunology, University of Montreal, CP 6128, Succursale Centre-Ville, Montreal, QC H3C 3J7, Canada; 4 Department of Life Science and Basic Science Institute for Cell Damage Control, Sogang University, Mapo, Shinsu 1, Seoul 121-742, Republic of Korea; 5 Division of Renewable Energy Engineering, University of Science and Technology, 217 Gajeong-ro, Daejeon, Yuseong-gu 305-350, Republic of Korea

**Keywords:** Photo-fermentative hydrogen production, Electron balance, Soluble microbial products, Ethanol, Lactate, Size exclusion chromatography

## Abstract

**Background:**

Biological fermentation routes can provide an environmentally friendly way of producing H_2_ since they use renewable biomass as feedstock and proceed under ambient temperature and pressure. In particular, photo-fermentation has superior properties in terms of achieving high H_2_ yield through complete degradation of substrates. However, long-term H_2_ production data with stable performance is limited, and this data is essential for practical applications. In the present work, continuous photo-fermentative H_2_ production from lactate was attempted using the purple non-sulfur bacterium, *Rhodobacter sphaeroides* KD131. As a gradual drop in H_2_ production was observed, we attempted to add ethanol (0.2% v/v) to the medium.

**Results:**

As continuous operation went on, H_2_ production was not sustained and showed a negligible H_2_ yield (< 0.5 mol H_2_/mol lactate_added_) within two weeks. Electron balance analysis showed that the reason for the gradual drop in H_2_ production was ascribed to the increase in production of soluble microbial products (SMP_s_). To see the possible effect of ethanol addition, a batch test was first conducted. The presence of ethanol significantly increased the H_2_ yield from 1.15 to 2.20 mol H_2_/mol lactate_added_, by suppressing the production of SMPs. The analysis of SMPs by size exclusion chromatography showed that, in the later period of fermentation, more than half of the low molecular weight SMPs (< 1 kDa) were consumed and used for H_2_ production when ethanol had been added, while the concentration of SMPs continuously increased in the absence of ethanol. It was found that the addition of ethanol facilitated the utilization of reducing power, resulting in an increase in the cellular levels of NAD^+^ and NADP^+^. In continuous operation, ethanol addition was effective, such that stable H_2_ production was attained with an H_2_ yield of 2.5 mol H_2_/mol lactate_added_. Less than 15% of substrate electrons were used for SMP production, whereas 35% were used in the control.

**Conclusions:**

We have found that SMPs are the key factor in photo-fermentative H_2_ production, and their production can be suppressed by ethanol addition. However, since external addition of ethanol to the medium represents an extra economic burden, ethanol should be prepared in a cost-effective way.

## Background

Hydrogen (H_2_), considered the cleanest energy alternative to fossil fuels, is now made exclusively by using fossil fuels: by steam reforming of natural gas, thermal cracking of light oil, and coal gasification. In contrast, biological fermentation routes can provide an environmentally friendly way of producing H_2_ since they use renewable biomass as feedstock and proceed under ambient temperature and pressure [1,2]. Biological H_2_ production occurs via two main pathways, classified as dark and photo-fermentation according to light dependency. Dark fermentative production has the advantage that the organic substrates are converted to H_2_ at a fast production rate, with, however, the disadvantage that volatile fatty acids are produced as side products, thus limiting total H_2_ yields [3,4].

Photo-fermentation is a process that can theoretically achieve maximum H_2_ yields through complete degradation of substrates [5]. Purple non-sulfur (PNS) bacteria have been the most intensively studied photosynthetic H_2_-producing bacteria due to their demonstrated high substrate conversion yields. Under illuminated anaerobic conditions, PNS bacteria can grow photoheterotrophically using organic substrates and can potentially produce H_2_. However, they have several alternative metabolic modes such as photoautotrophy, fermentation, and aerobic/anaerobic respiration, which are non-H_2_-producing or even H_2_-consuming pathways [6]. Therefore, it is critical to adjust the culture conditions so that they are suitable for H_2_ production.

Although PNS bacteria can use a wide variety of organic acids as a carbon source, the catabolic pathway involved depends on the substrate type. For example, acetate and butyrate are easily converted to acetyl units during metabolism by PNS bacteria and yield mainly polyhydroxyalkanoic acids (PHAs) rather than H_2_[7]. This leads to a decrease in H_2_ production, since PHA production is an undesirable electron sink in competition with photo-fermentative H_2_ production. Compared to acetate and butyrate, lactate and succinate are known to yield more H_2_[6,8]. Furthermore, since lactate can be easily obtained from anaerobic fermentation of agricultural and food wastes, it could be an ideal substrate for photo-fermentative H_2_ production [9].

Along with PHAs, soluble microbial products (SMPs) can also be an electron sink in photo-fermentation, competing with the H_2_ production pathway [10]. SMPs are soluble organic compounds that are released during biomass growth and cellular decay through normal metabolic processes [11,12]. Most studies on SMPs have been focused on aerobic systems, because SMPs often form the majority of the effluent chemical oxygen demand (COD) from biological treatment systems and are related to bio-fouling in membrane bioreactors (MBRs) [13,14]. However, there is little information about SMP production during photo-fermentation. Yilmaz et al. [10] quantitatively analyzed the partitioning of nutrient electrons into H_2_, cell biomass, PHAs, and SMPs in photo-fermentation by *Rhodobacter sphaeroides*. They reported that SMPs were a major electron sink, taking a large amount of reducing power away from H_2_ production. Similarly, Kim and Kim [15] found that 3% to 17% of substrate electrons were partitioned to SMP production during semi-continuous photo-fermentation, suggesting that SMPs are a major electron sink, taking large amounts of reducing power away from H_2_ production.

So far, numerous studies have been conducted to optimize photo-fermentative H_2_ production in batch-type operations, but there are only a few reports on continuous operation [16-18]. To our knowledge, long-term H_2_ production data with stable performance is limited; however, this data would be essential for the practical application of the process. In the present work, continuous photo-fermentative H_2_ production from lactate was attempted using the PNS bacterium *Rhodobacter sphaeroides* KD131. Electron balances were established to evaluate electron partitioning of lactate into H_2_, cell biomass, and SMPs. As a gradual drop in H_2_ production was observed during continuous operation, 0.2% (v/v) ethanol was externally added to the medium, since it has recently been shown that the presence of ethanol can enhance H_2_ production by *R. sphaeroides*[19]. In addition, size exclusion chromatography (SEC) and excitation-emission matrix (EEM) analysis were carried out to characterize the SMPs produced during photo-fermentation.

## Results and discussion

### Continuous performance

**Figure 1:**
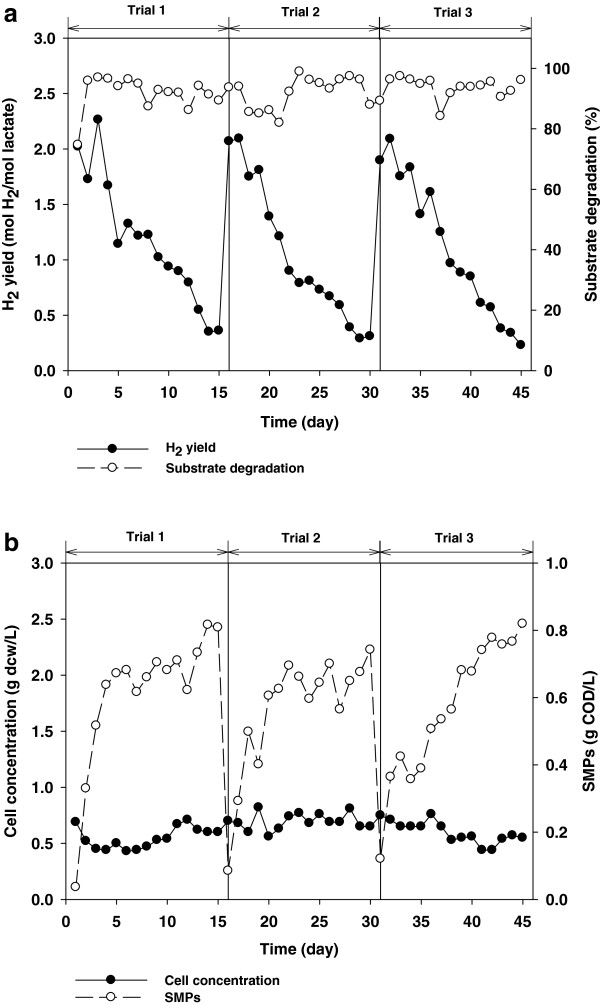
**Daily performance of photo-fermentative H**_**2 **_**production from lactate. (a)** H_2_ yield and lactate degradation, and **(b)** cell concentration and SMPs.

As shown in Figure 1a, H_2_ production from lactate was not sustained. H_2_ production gradually dropped and showed a negligible H_2_ yield (< 0.5 mol H_2_/mol lactate_added_) within two weeks. The theoretical maximum H_2_ yield from lactate is 6 mol H_2_/mol lactate_added_[7]. Although the fermenter was reseeded with a new inoculum (trials 2 and 3), the same phenomenon was observed. Unlike H_2_ production, lactate degradation and cell growth did not fluctuate during the entire operation period, suggesting that some portion of electrons contained in the substrate was diverted towards other paths (Figure 1b). Here, we have quantified the SMPs and demonstrate that changes in SMP concentration show an opposite trend to H_2_ production.

**Figure 2:**
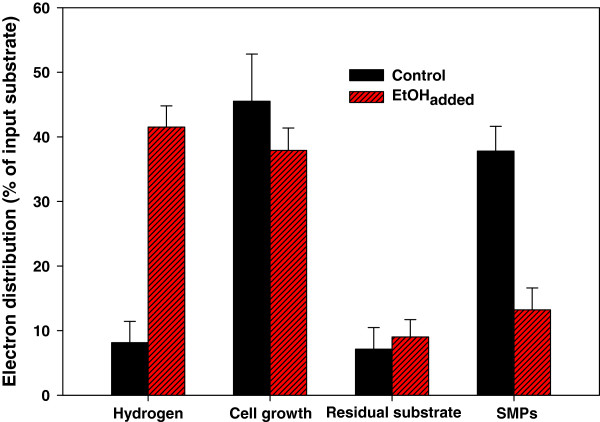
**Effect of ethanol addition on electron distribution in continuous photo-fermentative H**_**2 **_**production.** In the control (absence of ethanol), data from three independent experiments were collected after 10 days of operation and were averaged.

Establishing an electron balance is important in anaerobic fermentative processes, as it indicates the accuracy of the experiment and provides basic information regarding metabolic flux control [15,20,21]. In the present study, the COD was used as the common unit for electron balance, and the substrate and all metabolic products were converted to this unit. The electrons in the substrate that were utilized can be distributed to H_2_, cell growth, and SMPs. The result of this electron balance exercise can be seen in Figure 2. Unlike the work of Yilmaz et al. [10], in our study, PHA was not separately considered with cell growth since most of the PHA is retained inside the cell [22]. The amount of SMPs was measured by subtracting the residual lactate from the COD of samples that had been filtered through a 0.45-μm membrane. The COD of a cell was calculated by assuming a composition of C_5_H_7_O_2_N, resulting in a COD value of 1.42 g COD/g DCW (dry cell weight) [23]. While a fully accurate and precise electron balance was not possible since H_2_ production and SMP concentration fluctuated during the experimental period, it was informative to roughly determine electron distribution at the point of failure by collecting data at each trial after 10 days of operation.

The sum of metabolic products and the residual substrate accounted for 100 ± 10% of the input substrate, which indicates that the experiments were conducted with sufficient accuracy. From the electron balance obtained, a number of observations about the reasons for unsuccessful H_2_ production can be made. In the control, it would seem that most of the electrons were consumed for cell growth and SMP production rather than for H_2_ production. Around 45% and 38% of the electrons were diverted toward cell growth and SMP production, respectively. As the cell concentration did not vary during the continuous operation, the reason for the gradual drop in H_2_ production can be ascribed to the increase in SMP production as continuous operation proceeded.

### Effect of ethanol addition on batch-type fermentation

**Figure 3:**
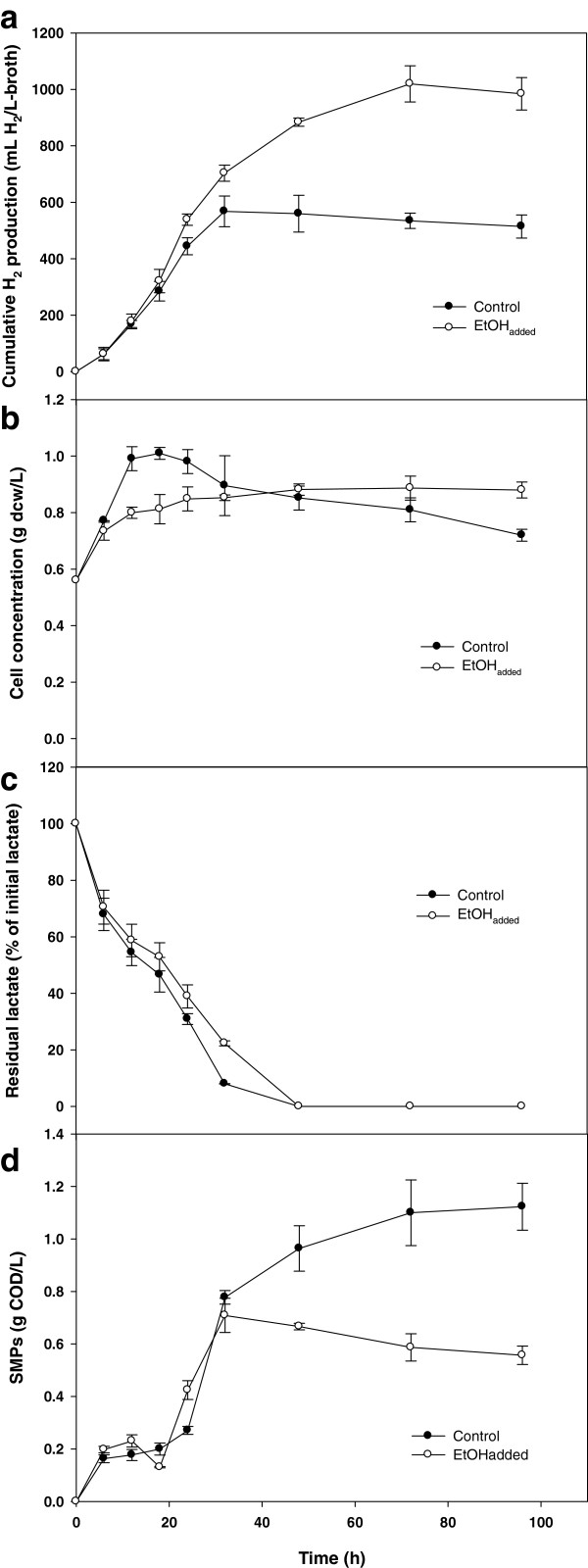
**Effect of ethanol addition on the batch performance of photo-fermentative H**_**2 **_**production from lactate. (a)** H_2_ production, **(b)** cell concentration, **(c)** residual lactate, and **(d)** SMPs.

We first assessed any possible effect of ethanol addition (0.2% v/v) by examining batch-type photo-fermentative H_2_ production (Figure 3). In the presence of ethanol, H_2_ production continued for 80 h while there was no more H_2_ production after 40 h in the control. Moreover, H_2_ yield was enhanced from 1.15 to 2.20 mol H_2_/mol lactate_added_ by the addition of ethanol (448 mL H_2_/L-broth = 1 mol H_2_/mol lactate_added_). There was not much difference in the profile of residual lactate concentration and pH (data not shown), but significantly different patterns were observed for cell concentration and SMP production depending on whether ethanol had been added or not. As operation continued, the pH gradually increased from 7.5 to 8.0 with lactate consumption. In the control (in the absence of ethanol), the cell concentration increased significantly until 20 h, and then gradually decreased afterwards. However, in the presence of ethanol, cell concentration continuously increased, finally reaching about a 20% higher cell concentration. In contrast, while SMP production continued for the entire experimental period in the control, it can be clearly seen that the addition of ethanol suppressed SMP production after 40 h. It is likely that the production of SMPs in the later periods of incubation resulted from microbial decay, since no further lactate degradation occurred after 50 h.

### Characterization of SMPs

**Figure 4:**
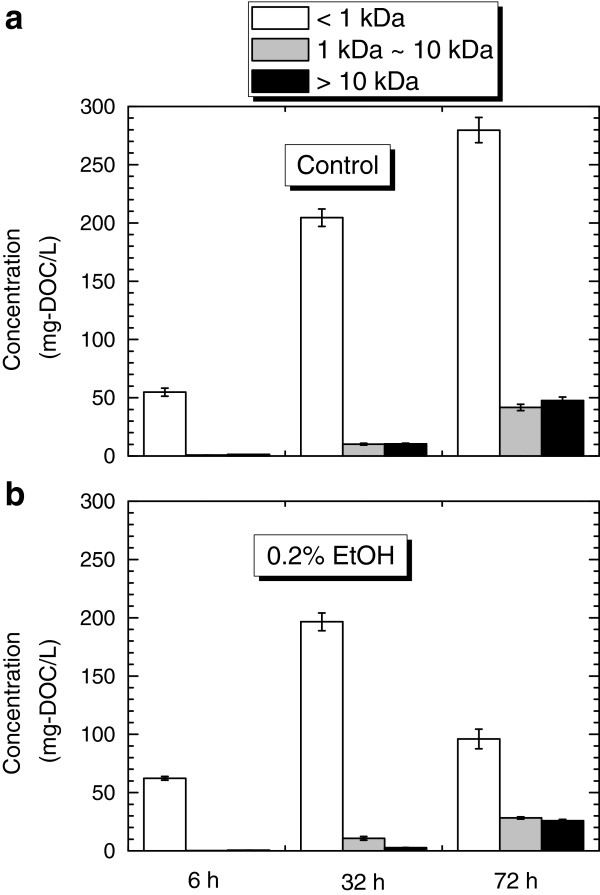
**The mass of organic matter (as dissolved organic carbon) according to molecular weight distribution of SMPs. (a)** in the absence (control), and **(b)** presence of 0.2% ethanol at 6, 32, and 72 h fermentation.

SMPs are defined as soluble organic compounds that are released during normal substrate metabolism (utilization-associated SMPs, UAPs) and decay in biological processes (biomass-associated SMPs, BAPs) [11]. UAPs are known to be further utilized by microorganisms, while BAPs are responsible for final effluent organic matter [11,24]. During batch photo-fermentation, it is likely that cell decay (or production of BAPs) became dominant after 40 h in the absence of ethanol, while additional conversion of SMPs to H_2_ took place in the presence of ethanol, as indicated by the data shown in Figure 4. Indeed, characterization of SMPs strongly supports this idea. The molecular weight distribution of SMPs analyzed at various incubation times showed that the mass of high molecular weight SMPs kept increasing during the incubation in both samples. However, the mass of low molecular weight SMPs (< 1 kDa) was significantly reduced from 197 mg dissolved organic carbon (DOC)/L at 32 h fermentation to 96 mg DOC/L at 72 h fermentation in the presence of 0.2% ethanol, while that of the control increased from 205 mg DOC/L to 280 mg DOC/L over the same time period. This supports the contention that the low molecular weight SMPs were converted to H_2_ in the presence of ethanol.

**Figure 5:**
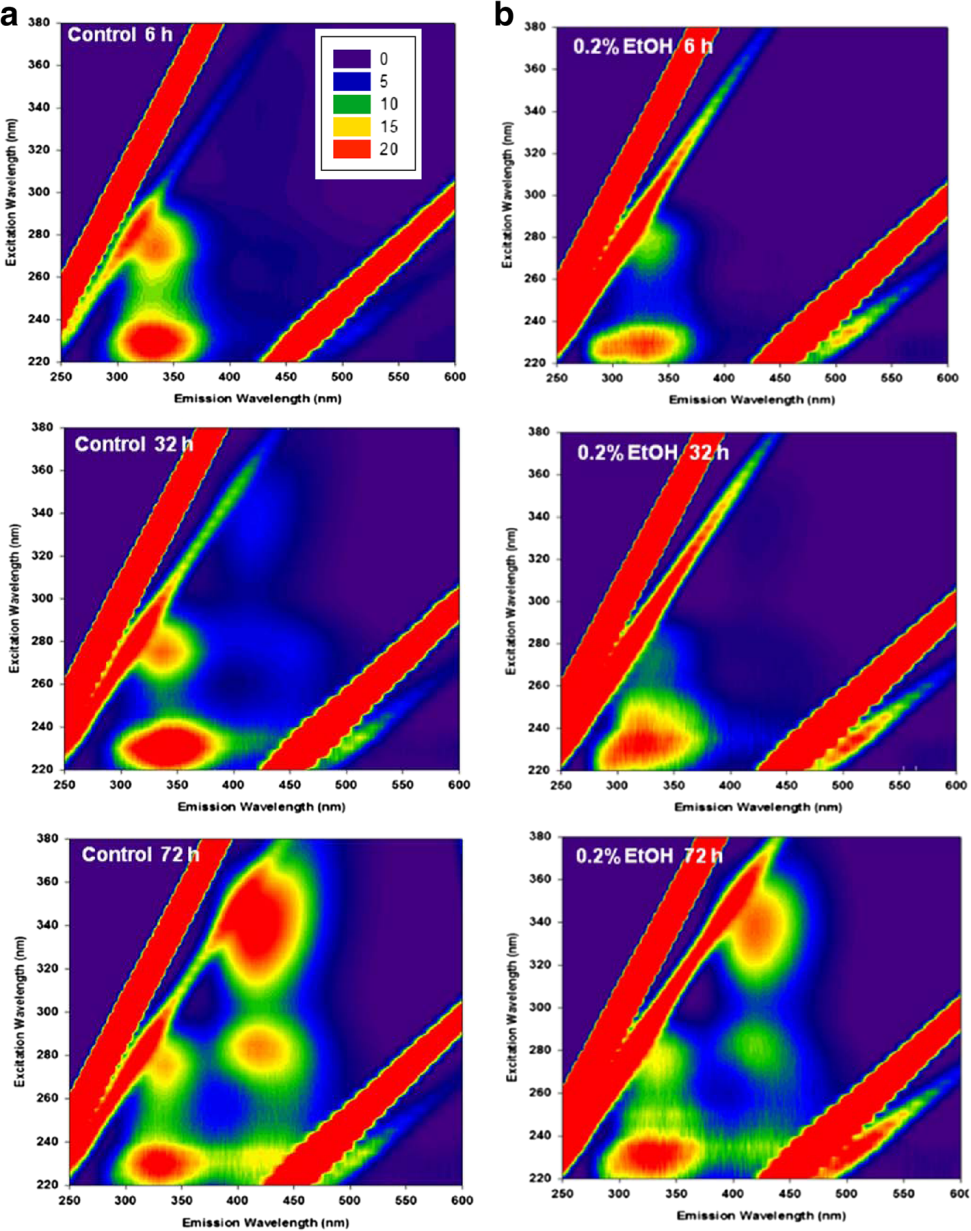
**Excitation-emission matrix of soluble microbial products. (a)** in the absence (control), and **(b)** presence of 0.2% ethanol after 6, 32, and 72 h fermentation.

In Figure 5, the EEM of SMPs taken at various fermentation times shows that the locations of peaks in the two samples are similar except for the peak with excitation at around 270 nm and emission at around 340 nm, thought to correspond to BAPs [25]. In the presence of ethanol, the BAP peaks were weaker than those of the control, indicating that the majority of SMPs produced in the presence of ethanol were mostly UAPs which could be further fermented to produce H_2_. After 72 h of incubation, at which point photo-fermentation is complete in both samples, the peaks responsible for high molecular weight SMPs (> 10 kDa) were revealed to be due to humic acid-like (excitation-emission at 320 to 360 nm and 400 to 450 nm) compounds which cannot be degraded by the microorganism. Compared to the control, these peaks were also weaker in the EtOH_added_ case, suggesting that the presence of ethanol can lead to less production of SMPs.

### Alteration of redox balance by the addition of ethanol

**Figure 6:**
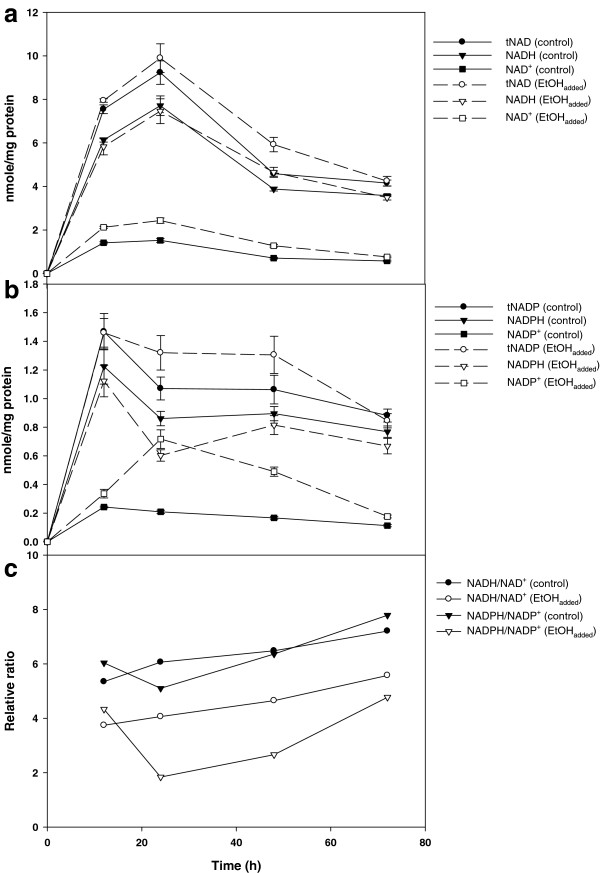
**Cellular level of NAD(H) and NADP(H) in the absence and presence of 0.2% ethanol of batch-type photo-fermentation. (a)** NADH, NAD^+^, and the total level of NADH + NAD^+^ (tNAD), **(b)** NADPH, NADP^+^, and the total level of NADPH + NADP^+^ (tNADP), and **(c)** the ratios of both NADH/NAD^+^ and NADPH/NADP^+^.

The reducing power for nitrogenase activity is provided by ferredoxin, which is reduced by NADPH/NADP^+^ (nicotinamide adenine dinucleotide phosphate):ferredoxin oxidoreductase [26]. Thus, the H_2_ production by nitrogenase is affected by the state of cellular reducing equivalents. To investigate the effect of ethanol on the equilibrium of cellular reducing equivalents, the cellular levels of NAD(H) (nicotinamide adenine dinucleotide) and NADP(H) were determined in the absence and presence of 0.2% ethanol. As shown in Figure 6a, NAD^+^ was increased by up to approximately 50% with the addition of ethanol, whereas the level of NADH was relatively unchanged by the presence of ethanol, in comparison with the case of the control. Interestingly, concomitant with the 10% to 30% decrease in NADPH, NADP^+^ increased almost threefold by the addition of ethanol (Figure 6b). Accordingly, the ratios of both NADH/NAD^+^ and NADPH/NADP^+^ were lowered to 40% to 70% of the control values that were determined in the absence of ethanol (Figure 6c). Since the nitrogenase activity of *R. sphaeroides* is elevated in the presence of ethanol [19], the consumption of reducing power appears to be accelerated by ethanol. Previously, an increase in the cellular NAD^+^ level was also observed following the deletion of nonessential metabolic pathways in *R. sphaeroides*[27]. When the metabolic flux leading to H_2_ production is increased, the equilibrium of cellular reducing equivalents appears to move towards a more oxidized state.

Excessive reducing power is generated during photosynthetic growth on reduced carbon compounds, and balance needs to be maintained through reductive pathways such as CO_2_ fixation, thereby yielding NAD^+^ and NADP^+^[28]. It has been suggested that, when growing heterotrophically under photosynthetic conditions, *R. sphaeroides* forms SMPs to remove excess reducing power [10]. As can be seen in Figure 6, it turns out that the NAD(H) and NADP(H) pools in the control photosynthetically grown *R. sphaeroides* still remain in the reduced state. Since BAPs were dominantly produced under photosynthetic conditions, the formation of BAPs is likely to result from this reduced state. On the other hand, when ethanol was added to the medium, the redox balance shifted to a more oxidized state, implying that more reducing power had been consumed by the cell. The level of BAPs was consistently decreased in the presence of ethanol. Moreover, UAPs, which were produced instead of BAPs in cultures exposed to ethanol, can be further utilized as a secondary fermentative organic source for H_2_ production. Thus, although excess reducing power can be eliminated by SMP formation (more specifically, BAP formation) during photo-fermentative growth of *R. sphaeroides*, more reducing power appears to be used up in the presence of ethanol, resulting in an increase of cellular NAD^+^ and NADP^+^.

### Effect of ethanol addition on continuous operation

**Figure 7:**
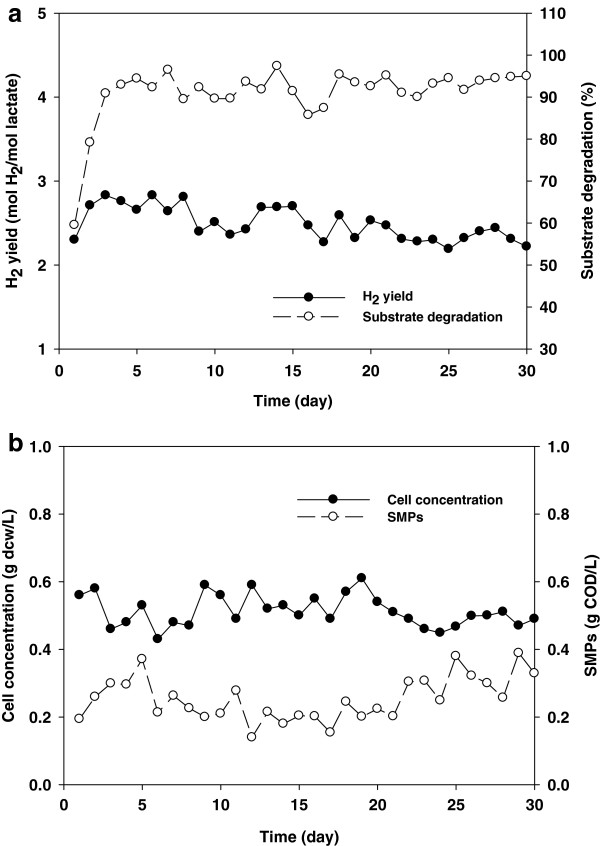
**Performance of continuous photo-fermentative H**_**2 **_**production from lactate in the presence of 0.2% ethanol. (a)** H_2_ production and substrate degradation and **(b)** cell concentration and SMPs.

Next we assessed the effect of ethanol addition on continuous photo-fermentative H_2_ production from lactate. Ethanol (0.2%) was added to the medium, which was continuously fed to the fermenter. As shown in Figure 7a, H_2_ production was stable for one month while giving an average H_2_ yield of 2.5 mol H_2_/mol lactate_added_ (corresponding to 41.6% of the total electron consumption). Lactate degradation and cell concentration were stable over this time period, and SMP production was minimized (Figure 7b). Less than 15% of electrons contained in the substrate were used for SMP production, whereas 35% were used in the control (Figure 2).

From the series of experiments, we found that SMPs are the key factor in photo-fermentative H_2_ production, and the SMP production can be suppressed by ethanol addition. However, since the external addition of ethanol to the medium represents an extra economic burden, ethanol should be prepared in a cost-effective way. For example, hetero-fermentative lactic acid bacteria ferment organic wastes to lactate and ethanol, which could be a suitable feedstock for photo-fermentative H_2_ production [29]. This process could be further developed by the optimization of operational parameters such as cell retention time, organic loading rate, and pH, all of which could influence SMP production.

## Conclusions

From the series of batch and continuous experiments on photo-fermentative H_2_ production from lactate in the absence and presence of ethanol presented here, the following conclusions can be drawn:

1. H_2_ production was not sustained during continuous photo-fermentative H_2_ production, as the H_2_ yield dropped lower than 0.5 mol H_2_/mol lactate_added_ within two weeks with increasing production of SMPs. Rather than H_2_, most of the electrons were diverted towards cell growth and SMP production.2. In batch operation, the presence of ethanol (0.2%) increased the H_2_ yield from 1.15 to 2.20 mol H_2_/mol lactate_added_ by suppressing the production of SMPs. Cell decay became dominant after 40 h in the absence of ethanol, while additional conversion of SMPs to H_2_ took place in the presence of ethanol. In the later period of fermentation, more than half of the low molecular weight SMPs (< 1 kDa) were consumed when ethanol had been added, whereas the SMP concentration continuously increased in the absence of ethanol. It was found that the addition of ethanol facilitated the utilization of reducing power, resulting in an increase in the cellular level of NAD^+^ and NADP^+^.

3. When ethanol was added during continuous operation, H_2_ production was stable for one month with an average H_2_ yield of 2.5 mol H_2_/mol lactate_added_ (corresponding to 41.6% of total electron consumption). Less than 15% of substrate electrons were used for SMPs production, whereas 35% were used in the control.

## Methods

### Inoculum preparation

The phototropic bacterium *R. sphaeroides* KD131, isolated from mud off the coast of Daebu Island in the West Sea of South Korea, was used for photo-fermentative H_2_ production. *R. sphaeroides* KD131 was pre-cultured in a modified Sistrom’s broth [30] containing 4 mM (NH_4_)_2_SO_4_, 0.3 mM L-aspartic acid, and 20 mM lactate at 30°C for 24 h under 110 W/m^2^ irradiance using halogen lamps (12 V, 50 W). The cells were capped in anaerobic tubes with O-rings and collected by centrifugation (8,000 rpm for 10 min, Supra 22 K, Hanil Co.) under anaerobic conditions, and used as an inoculum for H_2_ production.

### Experiments

For continuous operation, a 3.5-L glass fermenter (working volume of 3.0 L, 830 mm high by 80 mm in diameter) installed with a pH sensor at the top was used. Centrifuged biomass was added to reach an initial cell concentration of 0.56 g DCW/L equivalent to an optical density of 1.0. After purging with Ar gas (99.999%) for 1 h, the fermenter was operated for 48 h by batch mode as an adaptation period, and then switched to continuous mode. One liter of lactate (20 mM) containing medium (a modified Sistrom’s broth containing 4 mM (NH_4_)_2_SO_4_, and 0.3 mM L-aspartic acid) was continuously fed and removed per day, corresponding to three days of hydraulic retention time (HRT). During the operation, pH was maintained at 7.5 ± 0.2 by use of the pH sensor and the addition of 1 N HCl solution.

For the batch experiments to assess the effect of ethanol addition (0.2% v/v) on H_2_ production, 100 mL (effective volume of 50 mL) serum bottles were used, and the preparation procedure was the same as that used for continuous operation. The initial substrate concentration and cell concentration were 20 mM lactate and 0.56 g DCW/L, respectively. The amount of H_2_ production and the concentration of cell, residual lactate, and SMPs were measured periodically at intervals of 5 to 20 h. The experiment was conducted in triplicate and the results were averaged.

The light intensity (measured at the surface of the fermenter) was adjusted to 110 W/m^2^ using halogen lamps (12 V, 50 W), and all experiments were conducted in a temperature-controlled room at 30°C.

### Analytical methods

Measured biogas production was corrected to standard temperature (0°C) and pressure (760 mmHg) (STP). The H_2_ content in the biogas was analyzed using a gas chromatograph (Model 14-B, Shimadzu Co., Japan) equipped with a thermal conductivity detector and a stainless steel column packed with Molecular Sieve 5A (80/200 mesh; Altech, Deerfield, USA). 1 mL of sampled gas was injected, and the lower detection limit was 0.1%. The temperatures of the injector, detector, and column were kept at 100, 120, and 80°C, respectively. Organic acids were analyzed using a high performance liquid chromatograph (HPLC, Model VP, Shimadzu Co., Japan) equipped with a sulfonated divinyl benzene-styrene co-polymer column (300 mm × 7.8 mm, Aminex HPX-87H, BioRad, USA). 20 μL of sampled liquid was injected, and the lower detection limit was 0.01 mM. The column temperature was maintained at 35°C, and a photometric detector (216 nm) was used to quantify the organic acids eluted from the column. An aqueous solution of 10 mM H_2_SO_4_ was used as the eluting buffer and was dispensed at a flow rate of 0.6 mL/min. Liquid samples were pretreated with a 0.45-μm membrane filter (Millipore, USA) prior to injection to HPLC. The COD concentration was measured according to standard methods [31].

The supernatant filtered through a 0.45-μm membrane was regarded as SMPs after subtracting the residual lactate. After normalization of DOC to 3 mg DOC/L, the EEMs were examined using fluorescent spectroscopy (Shimadzu RF530, Japan) at excitation from 220 nm to 380 nm and emission from 250 nm to 600 nm. The EEMs were further normalized by using signals from water molecules as well as Rayleigh scattering, as previously suggested [25]. The average molecular weight of SMPs was analyzed by size exclusion chromatography using an HPLC (Younglin YL9101, Korea; Waters column, USA) with polysaccharides (dextran) as the molecular weight standard. The measurements of cellular NAD^+^, NADH, and their ratio as well as NADP^+^, NADPH, and their ratio were determined by using an NAD^+^/NADH quantification kit (BioVision, USA) and an NADP^+^/NADPH quantification kit (BioVision, USA), respectively.

## Abbreviations

BAPs: biomass-associated soluble microbial products; COD: chemical oxygen demand; DCW: dry cell weight; DOC: dissolved organic carbon; EEM: excitation-emission matrix; HPLC: high performance liquid chromatograph; HRT: hydraulic retention time; MBRs: membrane bioreactors; NAD: nicotinamide adenine dinucleotide; NADP: nicotinamide adenine dinucleotide phosphate; PHA: polyhydroxyalkanoic acid; PNS: purple non-sulfur; SEC: size exclusion chromatography; SMPs: soluble microbial products; STP: standard temperature and pressure; UAPs: utilization-associated soluble microbial products.

## Competing interests

The authors declare that they have no competing interests.

## Authors’ contributions

DHK performed the experiments, analyzed the results, and drafted the manuscript. JHL, SK, and EJK participated in the experiments. PCH and JKL commented on the manuscript. M-SK coordinated the study and revised the manuscript. All authors approved the final manuscript.
